# High‐Resolution Mapping of Strain Partitioning and Relaxation in InGaN/GaN Nanowire Heterostructures

**DOI:** 10.1002/advs.202200323

**Published:** 2022-06-05

**Authors:** Bumsu Park, Ja Kyung Lee, Christoph T. Koch, Martin Wölz, Lutz Geelhaar, Sang Ho Oh

**Affiliations:** ^1^ Department of Energy Science Sungkyunkwan University Suwon 16419 Republic of Korea; ^2^ CEMES‐CNRS 29 rue J. Marvig Toulouse 31 055 France; ^3^ Department of Physics Humboldt University of Berlin Berlin 12489 Germany; ^4^ Paul‐Drude‐Institut für Festkörperelektronik Leibniz‐Institut im Forschungsverbund Berlin e.V. Hausvogteiplatz 5–7 Berlin 10117 Germany; ^5^ Department of Energy Engineering KENTECH Institute for Energy Materials and Devices Korea Institute of Energy Technology (KENTECH) Naju 58330 Republic of Korea

**Keywords:** InGaN, light emitting diodes, nanowire heterostructures, strain, transmission electron microscopy

## Abstract

Growing an In*_x_* Ga_1−_ *_x_* N/GaN (InGaN/GaN) multi‐quantum well (MQW) heterostructure in nanowire (NW) form is expected to overcome limitations inherent in light‐emitting diodes (LEDs) based on the conventional planar heterostructure. The epitaxial strain induced in InGaN/GaN MQW heterostructure can be relaxed through the sidewalls of NW, which is beneficial to LEDs because a much larger misfit strain with higher indium concentration can be accommodated with reduced piezoelectric polarization fields. The strain relaxation, however, renders highly complex strain distribution within the NW heterostructure. Here the authors show that complementary strain mapping using scanning transmission electron microscopy and dark‐field inline holography can comprehend the strain distribution within the axial In_0.3_Ga_0.7_N/GaN MQW heterostructure embedded in GaN NW by providing the strain maps which can cover the entire NW and fine details near the sidewalls. With the quantitative evaluation by 3D finite element modelling, it is confirmed that the observed complex strain distribution is induced by the strain relaxation leading to the strain partitioning between InGaN quantum disk, GaN quantum well, and the surrounding epitaxial GaN shell. The authors further show that the strain maps provide the strain tensor components which are crucial for accurate assessment of the strain‐induced piezoelectric fields in NW LEDs.

## Graphical abstract

Comprehensive strain analysis by scanning transmission electron microscopy‐based geometrical phase analysis and dark‐field inline holography, in conjunction with 3D finite element modelling, demonstrates that strain relaxation through the sidewall induces the strain partitioning in the axial In_0.3_Ga_0.7_N/GaN multi‐quantum well heterostructure embedded in GaN nanowire. Furthermore, the piezoelectric polarization calculated using the measured in‐plane and out‐of‐plane strain indicates a significant piezoelectric field reduction compared to the planar heterostructure.

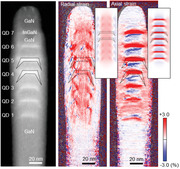

## 1 Introduction

The elastic strain arising in a heteroepitaxial thin film system can be utilized to enhance material's properties and functionality, for example, physical, electrical, and optoelectrical properties, which may differ from those of the bulk state. Representative examples of such strain engineering include Si metal‐oxide‐semiconductor field‐effect transistors where the carrier mobility is enhanced by inducing controlled epitaxial strain in the Si channels; and GaN‐based light emitters where the optoelectrical properties, for example, the emission wavelengths, are tuned through the manipulation of epitaxial strain.^[^ ^1^, ^2^, ^3^ ^]^ In particular, in In*_x_* Ga_1−_ *_x_* N/GaN (InGaN/GaN) multi‐quantum‐well‐structured light emitting diode (LED) devices, strain engineering is key to improve the device performance since the elastic strain can change the band structure of the quantum well (QW) through piezoelectric effects. The resulting piezoelectric fields are known to degrade the emission efficiency of III‐nitride LED devices.

While an InGaN/GaN LED for longer wavelength (green or red) emission requires high indium incorporation, the conventional planar heterostructures experience inherent growth problems such as the segregation of various phases^[^ ^4^ ^]^ and the formation of dislocations. This imposes a compromise between structural perfection and the target emission wavelength. To overcome this limitation of planar heterostructures, GaN nanowires (NWs) (or nanorods) with axial InGaN/GaN insertions have been suggested as a promising alternative. In contrast to the planar heterostructure where misfit strain is relieved by plastic deformation evolving defect formation above the critical thickness, the NW heterostructure allows elastic strain relaxation through free sidewalls that are sufficiently wider with respect to the volume, enabling defect‐free epitaxial growth even in the systems with a high indium concentration.^[^ ^5^, ^6^ ^]^ It has been theoretically^[^ ^7^ ^]^ predicted and experimentally^[^ ^8^ ^]^ verified that the elastic strain relaxation suppresses the piezoelectric polarization fields, which in turn leads to a higher internal quantum efficiency.

It is important to note that the InGaN layers in an InGaN/GaN multi‐quantum well (MQW) NW heterostructure are not grown simply in plate‐shape but mostly in truncated dome‐shaped quantum disk (QD) surrounded by the epitaxial GaN shell layer as well as GaN quantum barrier (QB).^[^ ^9^ ^]^ The QD shape of InGaN has been claimed on the basis of experimental HRTEM imaging.^[^ ^10^, ^11^ ^]^ Interestingly, it has been predicted by several theoretical studies that the InGaN QDs induce complex strain and polarization fields.^[^ ^12^, ^13^ ^]^ This further emphasizes the importance of comprehensive understanding on the correlation of the geometric shape and boundary condition of InGaN QD with the strain (and polarization) fields in InGaN/GaN MQW NW heterostructures.

While there is an increasing interest in controlling the optical properties of InGaN/GaN MQW heterostructures having complex 3D QD structures by understanding the detailed local strain distribution, conventional strain analysis methods (e.g., X‐ray diffraction analysis) provide only globally averaged information on the NW ensemble. In this regard, transmission electron microscopy (TEM)‐based strain mapping techniques can provide a unique solution with balanced combination of good spatial resolution, high strain sensitivity, and large field‐of‐view for individual NW heterostructures. There have been TEM studies investigating the strain distribution within InGaN/GaN NW heterostructures.^[^ ^9^, ^14^, ^15^, ^16^ ^]^ However, most strain maps, even though they successfully distinguish the different strain state of InGaN QDs from that of the GaN QBs, suffer from the high frequency noise obscuring the detailed strain distribution near the sidewalls where the InGaN QD shapes are delicately modified in correlation with strain relaxation or strain constraint by a surrounding GaN shell layer. Therefore, an experimental methodology that perfectly restores the full range of strain information in complex axial InGaN/GaN NW heterostructures is still missing.

In this study, we present the strain mapping of an axial In_0.3_Ga_0.7_N/GaN NW heterostructure by using two complementary methods which are high‐resolution scanning transmission electron microscopy (STEM) image‐based geometrical phase analysis (GPA)^[^ ^17^, ^18^, ^19^ ^]^ and dark‐field inline electron holography (DIH).^[^ ^20^, ^21^, ^22^ ^]^ We found that the InGaN QDs feature a truncated hollow dome shape, surrounded by an approximately 10 nm‐thick epitaxial GaN shell layer. The overall strain distribution within the NW heterostructure is reproduced well by the GPA of high‐resolution STEM images. We found that the compressive in‐plane strain in the InGaN QDs is reduced but the tensile in‐plane strain arises in the GaN QB due to the strain partitioning between the InGaN QD and the GaN QB. The DIH strain mapping technique allowed the details of the local strain distribution to be distinguished with much higher spatial resolution and strain sensitivity compared with the previous studies. The DIH in‐plane strain map shows that the strain in the GaN QB and the GaN shell is not uniform across the InGaN QD but tensile and compressive strain, respectively, alternates, i which is consistent with finite element method (FEM) calculations. We further show that the observed strain distribution is induced by the combined effects of strain relaxation and also constraining effects of the GaN shell layer. The piezoelectric polarization calculated using the measured in‐plane as well as out‐of‐plane strain indicates a significant reduction compared to that of a planar heterostructure.

## 2 Results and Discussion

### 2.1 Shape of InGaN QDs in Axial NW Heterostructure

**Figure 1:**
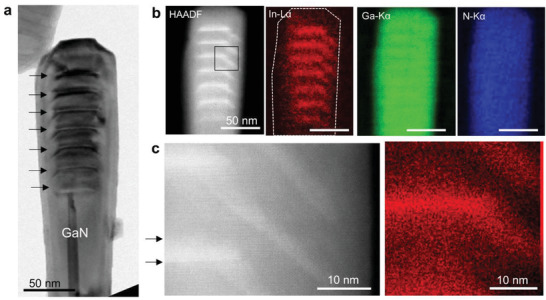
A model axial InGaN/GaN NW heterostructure. a) Cross‐sectional TEM bright‐field image showing the NW heterostructure. The MQW consisting of seven pairs of GaN QBs and InGaN QDs were formed between at least 400 nm‐long GaN NW at the base and 30 nm‐long GaN cap at the top. Seven dome‐shaped InGaN QDs are marked by arrows. b) STEM‐EDS elemental maps showing In, Ga, and N distribution in the InGaN/GaN heterostructure. c) Higher magnification STEM‐HAADF image and STEM‐EDS elemental map (In‐L_*α*_) of the InGaN QD edge (black box region in Figure 1b). Each InGaN QD has a truncated pyramidal shape with the $\left(000\bar{1}\right)$ central facet and the $\left\{10\bar{1}l\right\}$ side facets towards *m*‐plane sidewalls of the NW.

The axial InGaN/GaN NW heterostructure grown on Si (111) substrate (Figure S1a, Supporting Information) was characterized by STEM high‐angle annular dark‐field (HAADF) imaging and X‐ray energy dispersive spectroscopy (EDS) elemental mapping. The results summarized in **Figure** **1** show that the InGaN QDs have a truncated hollow dome shape with the $\left(000\bar{1}\right)$ central facet and the $\left\{10\bar{1}l\right\}$ side facets (*l* = 1, 2 or 3) towards the *m*‐plane sidewalls of the NW, which is similar to the faceted growth morphology of the NW tip. The lower interface of the InGaN QDs also adopts a similarly faceted shape. While the lower InGaN/GaN interfaces are atomically flat, the upper interfaces are rather diffuse and round (black arrows in Figure 1c). The InGaN QDs are completely overgrown by an epitaxial GaN shell, so that they are fully embedded within the GaN NW. With increasing number of the QD stack (excluding the first rather unstable one), the $\left\{10\bar{1}l\right\}$ side facets of the QDs become larger and steeper, reflecting the transition to smaller *l* indices (Figure S1b, Supporting Information). Similar QD structures have been observed in the previous studies.^[^ ^9^, ^11^ ^]^ Their origin is not understood in detail, but it is clear that the growth at NW tip provides a particularly complex framework for the interplay between strain, surfaces, and the immiscibility of (In,Ga)N, that is, well‐known relevant factors.^[^ ^10^, ^11^ ^]^ Along the same line, it is not surprising that in Figure 1c, indium atoms are also found in the GaN barriers as well. This poses questions on the growth mechanism and, more importantly, on the strain distribution within the heterostructures, as the elastic strain relaxation at the free sidewalls is impeded by the GaN shell.

### 2.2 Strain Field Simulation of NW Heterostructures with Various QD Shapes

In a planar lattice‐matched heterostructure only the adsorbate film is strained while the substrate and any grown layer of the same material remain unstrained. In an axial NW heterostructure, however, the strain distribution deviates from this simple picture due to the elastic strain relaxation at the free sidewall surfaces where all stresses go to zero. As a consequence of the elastic strain relaxation, the lattice coherency is maintained with varying extent of strain in adsorbate film and substrate material along the radial direction. According to the study of Wölz et al., the elastic strain relaxation results in a qualitatively similar strain field from the center of the NW to 80% of the radius.^[^ ^23^ ^]^ The elastic strain state of the axial NW heterostructure has been studied by analytical^[^ ^23^, ^24^, ^25^ ^]^ and numerical^[^ ^26^, ^27^, ^28^, ^29^, ^30^, ^31^ ^]^ calculations. Kaganer and Belov derived an analytical formulation for the nonuniform intrinsic strain distribution of stress‐free lattice parameter in an axial NW heterostructure with circular cross section.^[^ ^25^ ^]^ They implemented the analytical calculation by introducing an image strain field to the strain field produced by the intrinsic strain in an infinite medium (planar heterostructure) which is required to satisfy the cancellation of forces (stress‐free boundary conditions) on all free surfaces while imposing interfacial coherency.

**Figure 2:**
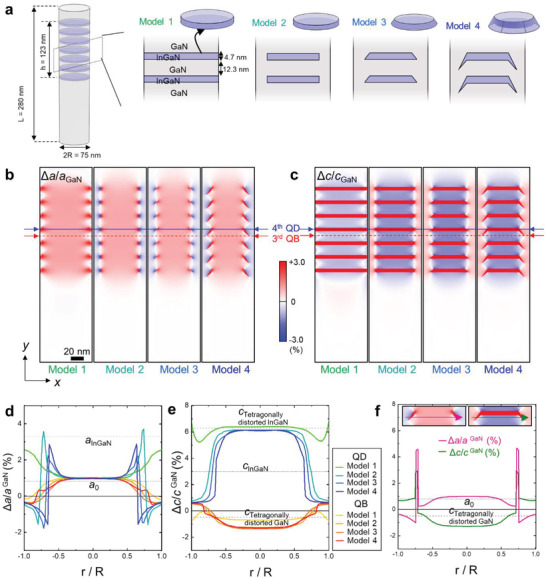
3D FEM strain simulation of model NW heterostructures with different QD shapes and boundary conditions. a) Schematics of the model NW heterostructures. b) 2D in‐plane strain maps and c) out‐of‐plane strain maps. The 2D strain maps were extracted from the central region of their respective 3D strain data. d,e) In‐plane strain profiles and out‐of‐plane strain profiles in the 4th InGaN QD (solid line in (b)) and the 3rd GaN QB region (dash line in (b)), respectively. The dash line indicated by *a*_0_ is the Δ*a*/*a* ^GaN^ calculated by using *a*_0_ = 3.214 Å from Equation (1). The dash line indicated by *a*_InGaN_ and *c*_InGaN_ is the Δ*a*/*a* ^GaN^ and Δ*c*/*c* ^GaN^ calculated by using the bulk lattice parameter of InGaN, respectively. The other two dash lines in (e) correspond to the Δ*c*/*c* ^GaN^ of InGaN (upper, labeled as *c*_Tetragonally distorded InGaN_) and of GaN (lower, labeled as *c*_Tetragonally distorded GaN_) calculated using the *a*_0_. The strain profile of the InGaN QD and the GaN QB in each model is color‐coded with gradually increasing blue tone and red tone, respectively, from model 1 to 4. f) In‐plane and out‐of‐plane strain profiles across the facet region of the InGaN QD in model 4.

The internal strain fields in an axial NW heterostructure depend critically on the thickness‐to‐diameter ratio, *h*/2*R*, where *h* is the thickness of the whole heterostructure and *R* is the radius of the NW. When *h* << 2*R*, the system is close to the planar heterostructure deposited on a semi‐infinite substrate and the elastic strain is essentially the same as in the laterally infinite system except at the very edge of the NW. As *h* increases, the QW starts relaxing strain in all directions (because top and lateral surfaces are free) and the substrate and epilayer share the strains even though the NW substrate is semi‐infinite. As a rule of thumb, this happens when *h* ≈ *2R*. When *h* increases further, any additional epilayer is deposited on an effectively strain‐free surface along the longitudinal direction and can thus adopt its own strain‐free state, so that the elastic energy does not increase any further. As a result, the axial strain of the NW substrate well above and well below the heterostructure (axial distance, z > 2*R*) is identically zero but localized within the hemispherical volume with the diameter of 2*R* (refer to the simulated strain maps in Figure 2b,c).

Considering the sufficiently large *h*/2*R* (≈1.64 with *h* = 123.2 nm and *R* = 37.5 nm) of the presented NW heterostructure and its effect on the strain relaxation, the misfit strain is shared between the InGaN QD and the GaN QB layers. Then, the solution for the strain partitioning becomes identical to the situation as in a laterally infinite free‐standing heterostructure without substrate and capping layer.^[^ ^25^ ^]^ For a strain partitioned free‐standing heterostructure, the equilibrium in‐plane lattice parameter (*a*_0_) can be calculated analytically by applying the following strain/force balance or energy minimization criterion:^[^ ^32^ ^]^

(1) $$a_{0}=\frac{A_{\mathrm{GaN}}t_{\mathrm{GaN}}a_{\mathrm{GaN}}a_{\mathrm{InGaN}}^{2}+A_{\mathrm{InGaN}}t_{\mathrm{InGaN}}a_{\mathrm{InGaN}}a_{\mathrm{GaN}}^{2}}{A_{\mathrm{GaN}}t_{\mathrm{GaN}}a_{\mathrm{InGaN}}^{2}+A_{\mathrm{InGaN}}t_{\mathrm{InGaN}}a_{\mathrm{GaN}}^{2}}$$

here, *A* defines the elastic constant for each layer. The elastic constants *A* are determined by the elastic stiffness coefficients, *C_ij_*, via the relation $A=C_{11}+C_{12}-2\left(C_{12}^{2}/C_{11}\right)$. The *C*_*ij*_ used for each layer is: *C*_11,GaN_ = 390 GPa and *C*_12,GaN_ = 145 GPa for GaN; *C*_11, InGaN_ = 339.9 GPa and *C*_12, InGaN_ = 136 GPa for In_0.3_Ga_0.7_N. *t* and *a* are the thickness and the bulk lattice parameter of each layer, respectively. The parameter *a*_0_ for the present NW heterostructure with 5 nm‐thick InGaN QDs and 12 nm‐thick GaN QBs is calculated to be *a*_0_ = 3.214 Å which is larger than the bulk GaN lattice parameter (*a*_GaN_ = 3.189 Å) but smaller than the lattice parameter of In_0.3_Ga_0.7_N (*a*_InGaN_ = 3.294 Å).

As a numerical approach to assess the detailed strain distribution, we applied the FEM within the framework of continuum elasticity theory. Four different model NW heterostructures were constructed with or without including a GaN shell layer and by varying the shape of QDs while keeping the thickness of InGaN QDs and GaN QBs constant (Figure 2a). The in‐plane strain (*ε_xx_*) maps in Figure 2b show that for all NW model structures the lattice coherency is achieved by complementary straining of the two layers, that is, compressive straining of InGaN and tensile straining of GaN, as a result of the strain partitioning. The in‐plane lattice parameter remains constant at *a*_0_ = 3.221 Å from the center to almost 40% of the cylinder radius (0.4*R*) (Figure 2b,d), which is comparable to the *a*_0_ = 3.214 Å calculated by Equation (1).

As a consequence of the strain partitioning, the out‐of‐plane lattice parameter of InGaN QDs is expanded less than what is expected for the planar heterostructure (note the green line in Figure 2e, which is denoted by tetragonally distorted InGaN). Furthermore, the GaN QBs are compressed along the out‐of‐plane direction (Figure 2c,e). We note that the FEM strain results are presented with respect to the GaN reference (the in‐plane strain as $\varepsilon_{xx}=\Delta a/a_{GaN}^{\mathrm{ref}}$, the out‐of‐plane strain as $\varepsilon_{zz}=\Delta c/c_{GaN}^{\mathrm{ref}}$) to compare with the experimental strain results. In the case of model 1 where the InGaN and GaN layers are extended all the way to the free sidewall, the strain distribution remains qualitatively similar to the one in the center up to ≈0.5*R*. From this position toward the surface, the in‐plane lattice parameters of InGaN and GaN are relaxed from *a*_0_ gradually towards their respective bulk value at the side surface, resulting in non‐uniform 3D strain. Once the in‐plane strain *ε_xx_* is determined, then the out‐of‐plane strain (*ε_zz_*) is simply assessed by the tetragonal distortion through the Poisson's effect, that is, *ε*_*zz*_ = −(2*ν*/1 − *ν*)*ε*_*xx*_, where *ν* is the Poisson's ratio. According to the FEM simulation result, the characteristic length of this surface relaxation, defined as an exponential decay length of the in‐plane strain profiles, is measured to be ≈10.5 nm. Interestingly, this characteristic length of surface relaxation remains similar in all four NW models constructed for FEM simulations.

A mixed heterostructure comprising an axial insertion of InGaN/GaN MQW and a surrounding GaN shell are treated in model 2. The surrounding epitaxial GaN shell blocks the strain relaxation of the InGaN QDs and GaN QBs and constrains their tetragonal distortion. As the lattice matching between the GaN shell and the InGaN QDs is established along the out‐of‐plane direction with strain partitioning, the GaN shell is tensile‐strained along the out‐of‐plane direction and consequently compressed along the in‐plane direction through the Poisson effect. As a consequence, there exists a singularity in the in‐plane strain at the interface between the InGaN QD and GaN shell, across which the sign of in‐plane strain is reversed. We recall that the stress‐free boundary conditions are effective within the characteristic length from the side surface; the GaN shell/InGaN QD interface lies within the characteristic length from the surface, thus it does not effectively affect the strain in the core region of NW.

Faceting of the vertical InGaN QD edge toward a $\left\{10\bar{1}l\right\}$ plane (model 3) does not change the overall strain pattern. The strain singularity appears along the slanted GaN shell/InGaN QD interface. In the hollow dome shape of the InGaN QD (model 4), which corresponds to what we observed in the experiment, the InGaN faceted edge region is surrounded by the GaN shell and the GaN QB with the opposite sign of strain (Figure 2f). The strain within this confined volume of InGaN facets changes steeply across the interface with GaN, indicating a large strain gradient which, in turn, induces a large piezoelectric polarization change.

### 2.3 Strain Field Measurements of NW Heterostructure

**Figure 3:**
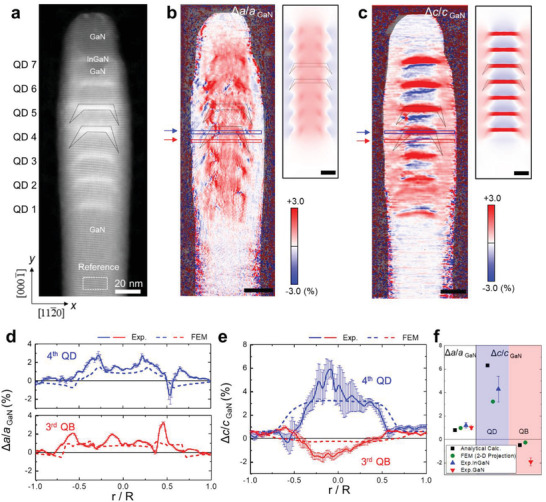
STEM‐GPA strain mapping results. a) STEM HAADF image of selected NW. The zone axis corresponds to the $\left[1\bar{1}00\right]$. (b) and (c) in‐plane and out‐of‐plane strain map, respectively. For comparison, the projected FEM strain map of the corresponding model NW heterostructure (Model 4 in Figure 2) is shown next to the experimental strain map. d) In‐plane strain profiles and e) out‐of‐plane strain profiles along the 4th InGaN QD (blue box in (b,c)) and the 3rd GaN QB region (red box in (b,c)). Solid and dotted profiles correspond to experimental results and averaged FEM results, respectively. f) Measured in‐plane and out‐of‐plane strain values averaged near the central region (−0.1 *R* < *R* < 0.1*R*). For comparison, the strain expected by the analytical calculation (Equation (1), black square) and the FEM simulation (green circle) are also indicated.

For strain mapping of the whole NW heterostructure, large‐area high‐resolution STEM HAADF images covering the entire heterostructure were prepared by stitching the images obtained locally from the GaN foot to the GaN cap of the NW with a sampling of 4k × 4k pixels. Then, the unstrained GaN foot region was used as the reference for the strain measurement of all regions. Figure 3a shows a typical STEM HAADF image prepared for strain mapping. The in‐plane and the out‐of‐plane strain maps obtained by GPA of the STEM image are shown in Figures 3b,c, respectively. We remind once again that the strain map is a fractional change in the lattice parameters with respect to bulk GaN, that is, $\varepsilon_{xx}=\Delta a/a_{GaN}^{\mathrm{ref}}$ and $\varepsilon_{zz}=\Delta c/c_{GaN}^{\mathrm{ref}}$. For comparison with the experimental strain maps, the 3D strain maps simulated by FEM (corresponding to model 4 in Figure 2) were averaged along the electron beam direction (Figure S2, Supporting Information). Except for slight differences in fine details which arise mainly from the different QD shape in simulation and experiment, the key features observed in the experimental in‐plane strain map agree qualitatively well with the simulated strain maps, which are: the positive in‐plane strain (*ε_xx_* > 0) in the central region of heterostructure due to strain partitioning between InGaN QD and GaN QB and the negative in‐plane strain (*ε_xx_* < 0) in the GaN shell due to the lattice matching on the inclined facet of the InGaN QD. Similarly, the out‐of‐plane strain $\left(\varepsilon_{zz}=\Delta c/c_{GaN}^{\mathrm{ref}}\right)$ in the InGaN QD, GaN QB, and GaN shell region of the experimental result are also in good agreement with the corresponding region of the FEM simulation.

To evaluate strain partitioning and relaxation, the strain profiles were extracted from the experimental strain maps and compared with the FEM results (Figure 3d,e and Figure S3, Supporting Information). The experiment and the FEM result is represented by solid and dash line, respectively. The averaged *ε_xx_* of the InGaN QD and the GaN QB in the central region (−0.1*R* < *x* < 0.1*R*) are similar, which is 1.2 ± 0.12% and 1.02 ± 0.19%, respectively, confirming the strain partitioning between the two layers (according to the conventional definition of strain, the measured strain corresponds to −2.29% for the InGaN QD and 1.02% for the GaN QB). The analytical calculation using Equation (1) and the FEM simulation for the GaN QB yields 0.80% and 0.98%, respectively, which agree well with the measured value. The in‐plane lattice parameter converted from the strain of InGaN QD and GaN QB yields *a*_InGaN QD_ = 3.227 Å and *a*_GaN QB_ = 3.222 Å, respectively. Moving from the central region to the GaN shell, the in‐plane strain of the InGaN QD rises and falls across the interface with the GaN shell. Although both profiles of experiment and simulation are qualitatively similar, the experimental profile shows larger strain variation across the InGaN QD/GaN shell interface. This is due to the different volume fraction of InGaN and GaN along the projection direction as the shape of InGaN QDs is likely different from the circular shape assumed in the FEM simulation. The compressive in‐plane strain (−0.3 ± 0.2%) of the GaN shell, which originates from the strain partitioning with the InGaN QD, agrees well with the FEM simulation (−0.5%). The in‐plane strain of the GaN shell is predicted to increase gradually from the InGaN QD/GaN shell interface toward the bulk GaN value in the FEM simulation. However, such a subtle change is not clearly resolved in the experimental strain map due to the limited strain sensitivity and the background noise inherent in the GPA method of strain mapping using STEM image.

The lateral profiles of the out‐of‐plane strain maps clearly show that the GaN QB and the GaN shell are compressed and expanded, respectively, as a consequence of strain partitioning with the adjacent InGaN QD (Figure 3e). In the central region (−0.1*R* < *x* < 0.1*R*), the averaged out‐of‐plane strain of the InGaN QD and the GaN QB is measured as 4.3 ± 1.1% and −1.9 ± 0.33%, respectively (Figure 3f) (note that, for comparison, the out‐of‐plane strain $\varepsilon_{zz}=\Delta c/c_{GaN}^{\mathrm{ref}}$ expected for the corresponding InGaN and GaN layers in a planar MQW structure without strain partitioning is 7.37% and 0%, respectively). While the experimental and simulated profiles agree qualitatively well, the former, accompanied by large local fluctuations (Figure 3e), tends to overestimate the average strain of both InGaN QD and GaN QB layers (Figure 3f). Such discrepancy may arise from the finite depth‐of‐field of the STEM probe (≈30 nm) which measures the strain over a finite volume of the InGaN QD while the FEM strain is averaged throughout the whole region. The fluctuation may also be caused by local variation in indium concentration or the background noise.

In the case of DIH it is not easy to obtain a reliable strain map covering the entire NW area because over a large area it is more likely to be affected by the local bending of the specimen, for example, those induced by defects, and the limited capability of the technique to recover very low spatial frequency information. A typical example of the through‐focal series of TEM dark‐field images obtained for strain mapping is shown in Figure S4, Supporting Information, and its reconstructed amplitude and phase images are shown in Figure S5, Supporting Information. As a further verification of the measured strain partitioning and relaxation, we obtained the strain maps using 4D‐STEM (Figure S6, Supporting Information).^[^ ^33^, ^34^ ^]^ While the overall strain features distinguishing the InGaN QDs from the GaN QBs and the GaN shell layer observed in the FEM simulation are reproducible, it is difficult to resolve the fine details in the vicinity of InGaN QDs due to the limited spatial resolution of the 4D‐STEM data (≈2 nm), which originates from various sources such as: the probe diameter (≈0.2 nm), the interface broadening due to a finite convergence angle (≈1 nm), and the local beam tilt by sample bending (≈0.7 nm). The thickness of the InGaN QD is very small, ≈5 nm, and just 1 pixel shift of the diffraction disc in the in‐plane direction causes a large strain difference. Also, the sensitivity of the 4D STEM strain value is affected by the pixel size of the detector. As such, the 4D STEM results are limited in providing reliable strain information for the fine details of NW heterostructure (the spatial resolution is even inferior to that of STEM‐GPA). However, the 4D STEM strain maps are able to visualize the overall strain relaxation effects on the strain distribution in the NW heterostructure.

### 2.4 Strain Distribution near the Side Surface

**Figure 4:**
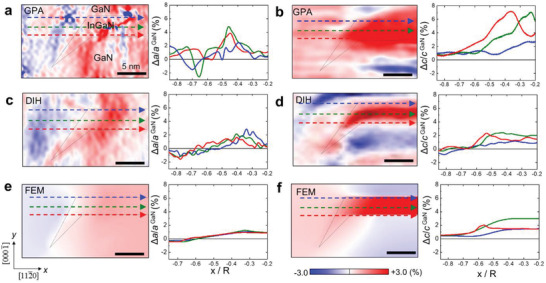
Comparison of the strain distribution near the side facet of InGaN QD obtained by different methods. a) In‐plane and b) out‐of‐plane strain map and 1D profiles obtained by the STEM‐GPA c,d) DIH and e,f) FEM, respectively. The strain data are compared by the profiles drawn along: InGaN/GaN lower interface (red line), middle position of QD (green line), and GaN/InGaN upper interface (blue line). As the STEM‐GPA signal is dominated by high frequency noise, it is difficult to extract meaningful strain information which can be compared with FEM results. On the other hand, DIH shows the strain profiles which match well with not only the overall variation across the different materials but also quantitative values.

While the overall strain distribution in the NW heterostructure is reproduced well by applying GPA to high‐resolution STEM images, detailed strain features in local regions are not clearly resolved due to the high‐frequency noise inherent in this method. While the spatial resolution of the STEM‐GPA strain map may be increased by applying a relatively large mask in reciprocal space, this also increases noise.^[^ ^17^, ^18^, ^19^ ^]^ Since the GPA interprets the positions of the maxima in image contrast as atom positions, this method can be fooled by defocus, aberrations of the probe‐forming optics, and also system instabilities; thus noise will be enhanced, especially, since the reported strain is computed as the gradient of the displacement signal being recovered. Especially, the strain distribution near the sidewall surfaces where strain relaxation is sensitively influenced by the presence of a surrounding epi‐layer and the shape of the QD is obscured by this noise. For example, in the in‐plane strain map shown in Figure 4a, the compressive strain in the GaN shell is distinguished from the tensile strain in the InGaN/GaN MQW but severely perturbed by the noise. Furthermore, due to the presence of large background noise in the strain map of STEM‐GAP the strain is likely to be overestimated by a few percentage, as illustrated by the strain profiles in Figure 4a; sharp peaks appear in the strain profiles of the STEM GPA strain results, which do not exist in the simulated strain map (Figure 4e), nor the DIH result (Figure 4c). These peaks are due to the mixing of Z‐contrast and strain that occurs when computing the gradient of the geometric phase extracted from STEM HAADF image, since the InGaN atomic columns have a stronger contrast than the GaN columns.

**Figure 5:**
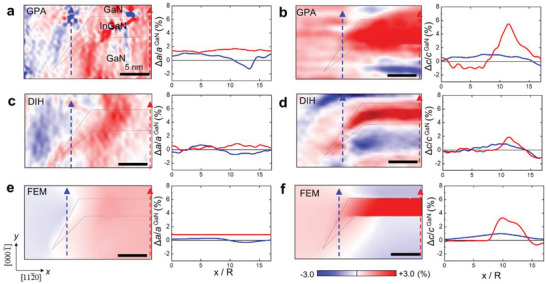
Comparison of the strain distribution near the side facet of InGaN QD obtained by different methods. a) In‐plane and b) out‐of‐plane strain map and 1D profiles obtained by the STEM‐GPA c,d) DIH and e,f) FEM, respectively. The three strain data were compared by the profiles drawn across: the central region of InGaN QD (red line), the central region of the inclined InGaN facet (blue) along the axial direction. While the DIH shows the strain profiles matching well with FEM results not only the overall variation across the different materials but also quantitative values, the high frequency noise in STEM‐GPA signal results in the overestimation of strain values.

DIH strain mapping is based on the geometric phase reconstruction from a through‐focal series acquired with a selected diffracted beam (Figure S4, Supporting Information). Owing to the good transmission of high spatial frequency information (Figure S7, Supporting Information), the method can provide reliable strain distribution of local regions with high sensitivity, which is well suited for the evaluation of the complex strain relaxation in the InGaN facet region near side surfaces. The DIH method which does not require atomic resolution data and compensates for contrast transfer function (CTF) induced delocalization, detailed strain distributions can be obtained at intermediate length scales, such as the fine structure of the NW. Figures 4c,d show the in‐plane and out‐of‐plane strain maps obtained by the DIH method, which reproduce perfectly the key features of the simulated strain map. In addition, the strain profiles extracted from the DIH strain maps match the simulated profiles in a quantitative manner. For example, the horizontal and vertical profiles drawn across the GaN shell and the InGaN QD in Figures 4c,d and 5c,d, respectively, reveal almost identical strain distribution to the simulated strain.

Since the results of DIH strain mapping are 2D projection results, it is difficult to isolate the strain distribution of the InGaN facet alone due to the continuous change of the projected volume fraction of GaN. However, the inversion of the sign of strain across the interface between InGaN facet and GaN shell is clearly observed in the DIH in‐plane strain map (Figure 4c). In addition, the 1D profiles of the DIH in‐plane strain map obtained along the lateral direction at the upper and lower interfaces, and middle position of the QD show almost similar to each other (Figure 4c), which also agree well with those of FEM results (Figure 4e). On the other hand, the DIH out‐of‐plane strain profiles obtained along the corresponding locations are clearly different to each other (Figure 4d); a peak strain exists at the lower facet interface (red line), while the strain increases at middle position (green line), and the strain remains the smallest near the upper interface (blue line), which are also in good agreement with those of FEM results (Figure 4f).

The vertical strain profiles drawn along the axial direction clearly show that the strain near the side surface (blue line in Figure 5) is reduced compared to that from the inner region (red line) as a consequence of strain relaxation. In addition, the inversion in the sign of in‐plane strain induced by the presence of GaN shell is reproduced for the GaN QB and the GaN shell (Figure 5a–c). While the DIH shows the vertical strain profiles matching well with FEM results not only the overall variation across the different materials at different radial locations but also quantitative values, the high frequency noise in STEM‐GPA signal results in the overshooting of strain values, for example, the blue line in Figure 5a and the red line in Figure 5b.

As demonstrated by the DIH strain mapping, the GaN shell modifies the strain distribution in the InGaN QDs by constraining the strain relaxation. Actually, the surrounding GaN shell makes the InGaN QDs fully embedded within GaN. As a consequence, the strain partitioning between the InGaN QDs and the surrounding GaN shell and QBs reduces the overall strain in the InGaN QDs. In addition, the surrounding GaN shell and QBs are also strained with opposite sign, which modifies the distribution of piezoelectric polarization and associated polarization charges at the interfaces.

### 2.5 2D Piezoelectric Polarization Mapping

The strain fields around the InGaN QDs modify the wave function of charge carriers and the electronic band structure.^[^ ^13^, ^28^, ^29^, ^35^ ^]^ Not only the strain‐band structure coupling but also the piezoelectric effects can significantly change the electronic and optical properties.^[^ ^36^ ^]^ Thus, the strain partitioning and relaxation in an axial NW heterostructure is expected to alter the piezoelectric polarization, associated charge distribution, and in turn internal quantum efficiency.^[^ ^15^, ^16^, ^37^, ^38^ ^]^ In general, strain relaxation occurring in the NW heterostructure is known to reduce the piezoelectric field and thus enhance electron–hole overlap and, consequently, the internal quantum efficiency. This relation is very well known and has been addressed in many previous publications.^[^ ^15^, ^16^, ^37^, ^38^ ^]^

Using the 2D strain maps obtained by STEM‐GPA and DIH, we calculated the piezoelectric polarization (*P_i_*) induced along the direction *i* by the strain *ε_j_* along the direction *j* through *P_i_* = ∑*e_ij_ε*_*j*_, where *e_ij_* is the piezoelectric coefficient. Assuming the isotropic in‐plane strain condition, the component of the polarization vector along the *z* direction, *P*_3_, was calculated using the following relationship:

(2) $$P_{3}=2e_{31}\varepsilon_{1}+e_{33}\varepsilon_{3}$$

where *ε*_1_ ( = *ε_xx_* = *ε_yy_*) is the in‐plane strain and *ε*_3_ ( = *ε_zz_*) is the out‐of‐plane strain for each layer of the heterostructure. The values *ε*_1_ and *ε*_3_ were derived from the measured *ε_xx_* and *ε_zz_* strain maps, respectively (the *ε_xx_* and *ε_zz_* of InGaN was converted to the actual strain by relating them to its bulk lattice parameters). The piezoelectric coefficients, *e_ij_*, used in the calculation are: *e*_33_ = 0.73 C m^−2^, *e*_31_ = −0.34 C m^−2^ for GaN, and *e*_33_ = 0.802 C m^−2^, *e*_31_ = −0.664 C m^−2^ for InGaN with 30% of indium.^[^ ^39^ ^]^

**Figure 6:**
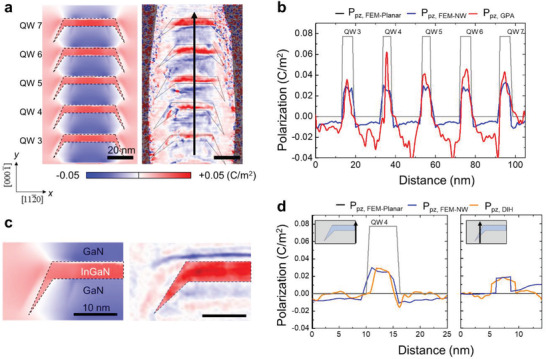
2D piezoelectric polarization maps. a) 2D piezoelectric polarization map calculated by using in‐plane and out‐of‐plane strain maps of 3D‐FEM (left) and STEM‐GPA (right). b) Piezoelectric polarization profile (*P*_3_, red solid line) obtained in the center area along with axial direction, which is indicated in (a) by black arrow. The measured piezoelectric polarization profile is compared with the FEM results of the planar structure (gray solid line) and NW structure (blue solid line). c) Comparison of magnified FEM piezoelectric polarization map (left) and polarization map obtained by DIH (right). d) 1D piezoelectric polarization profile obtained at the center of NW (left) and the InGaN facet (right) along the axial direction. 3D FEM results from planar structure (gray solid line) and NW structure (blue solid line) are included for comparison with DIH results from NW (orange solid line).

The map of *P*_3_ obtained by using FEM and STEM‐GPA strain data are shown in Figure 6a (left: FEM, right: STEM‐GPA), respectively. The key features of the polarization pattern are quite similar in the two maps; for example, the *P*_3_ with opposite sign is induced in the InGaN QDs (positive) and the GaN QBs (negative) as a consequence of strain partitioning. In Figure 6b the line profiles of *P*_3_ drawn from the central region of heterostructure are compared with that of the planar heterostructure with the same multiple InGaN/GaN stack. Compared to the planar heterostructure, the positive *P*_3_ value of the InGaN QDs is reduced considerably but an additional negative value of *P*_3_ arises in the GaN QBs: The calculated *P*_3_ *_,_*_InGaN_ = 0.0776 C m^−2,^ *P*_3_ *_,_*_GaN_ = 0 C m^−2^ for planar structure and *P*_3_ *_,_*_InGaN_ = 0.0412 C m^−2,^ *P*_3_ *_,_*_GaN_ = −0.003 C m^−2^ for hollow dome QDs system. On the other hand, the GaN shell attains a positive value of *P*_3_ due to the strain partitioning with InGaN QDs across the inclined interface, which is the same sign as that of the InGaN QDs. According to ∇ · *P*_3_ = −*ρ*, the gradient of *P*_3_ induces the fixed polarization charge *ρ* at the InGaN/GaN interfaces. Compared to the planar heterostructure, the polarization charge at the InGaN QD/GaN QB interface of the NW heterostructure is reduced as consequence of strain partitioning.

There are some noticeable differences between the polarization profiles of the FEM model and the experiment. The *P*_3_ polarization of each InGaN QD and GaN QB remains constant in the FEM model but this is not the case in the experiment; along the growth direction the experimentally measured polarization tends to increase both in the InGaN QD and GaN QB from the bottom to the top, reaching the largest value in the middle of the heterostructure. Obviously, this trend is closely related with the strain distribution which shows the largest strain value in the corresponding region of the heterostructure. We may hypothesize several causes for this trend. Considering the high STEM HAADF intensity of the InGaN QDs in the middle of heterostructure (Figure 3a), one may speculate a higher indium content in the InGaN QDs, which can modify the strain as well as *e_ij_*. In addition, the non‐uniform diameter of the NW heterostructure, especially the tapering of the GaN cap and foot regions, that is, a barrel‐like shape, may be related to a relatively large extent of strain relaxation. We also note that the depth‐of‐field in high‐resolution STEM HAADF imaging is reduced by the high convergence angle required to form a fine probe. With a depth‐of‐field value of ≈30 nm, it is smaller than the diameter of the NW, sampling predominantly a slice of the NW volume.

The polarization in the GaN shell region is not well reproduced by the STEM‐GPA method due to the noise and the limited depth‐of‐field of the corresponding strain maps. As shown in Figure 6c, the *P*_3_ polarization map obtained by using the DIH strain data reveals the detailed polarization near the facet of InGaN QDs more reliably. The 1D profile obtained in the central region shows that the DIH and FEM strain results not only match well with respect to the key features but also the quantitative polarization values. In particular, the facet region seems to show more reduction in the polarization field due to strain relaxation compared to the central region (Figure 6c,d). The decrease in the polarization field in the InGaN QDs is more pronounced in the cross‐sectional polarization field map (Figure S8, Supporting Information). However, one should pay attention to the polarization gradient across the side facet of InGaN QDs (NW model 4 in Figure S8c, Supporting Information); the negative *P*_3_ of the GaN QB changes to the positive values sequentially in the InGaN QD and the GaN shell, of which gradient is quite different from other model systems. As a consequence, the same sign of polarization charges with reduced magnitude are induced at the upper and the lower InGaN/GaN interfaces of the InGaN facet. This interface charge distribution leads to a relatively flat band in the InGaN QD facet region, which may enforce the internal quantum efficiency (Figure S8d, Supporting Information).

It is interesting to note how the reduced piezoelectric fields by strain relaxation affect the luminescence of InGaN/GaN NW heterostructure compared to conventional planar heterostructures. As illustrated in Figure S8d, Supporting Information, the reduced piezoelectric field alone is expected to enhance the internal quantum efficiency by increasing the overlap between electron and hole wave functions. However, the light emission from NW heterostructure is influenced by various effects in addition to piezoelectric fields, such as the volume of active material, light coupling, competing non‐radiative recombination, and diffusion of charge carriers. Therefore, a meaningful comparison of light emission from NW and equivalent planar heterostructures would be a complex study in its own right that would go far beyond our current analysis focusing on strain and the resulting piezoelectric fields.

## 3 Conclusion

Taking an axial InGaN/GaN NW heterostructure grown by a bottom‐up approach as model system, we investigated the strain distribution within the NW heterostructure by applying two complementary strain mapping techniques. The InGaN QDs feature a faceted hollow dome shape, surrounded by an approximately 10 nm‐thick epitaxial GaN shell layer. To quantitatively evaluate the strain partitioning and relaxation, FEM strain mapping was carried out in a systematic way by changing the geometry and shape of the InGaN QDs and also with including the GaN shell. The overall strain distribution within the NW heterostructure is reproduced well by the GPA of high‐resolution STEM images. Due to the strain partitioning between the InGaN QD and the GaN QB, the compressive in‐plane strain in InGaN is reduced but the tensile strain arises in the GaN. The DIH strain mapping allowed the details of local strain distribution to be distinguished with much higher spatial resolution and strain sensitivity. The DIH in‐plane strain map shows that the strain across the InGaN QDs is not uniform but tensile and compressive strain alternates in the GaN QB and the GaN shell, respectively, which is consistent with FEM calculations. In addition, while exhibiting almost similar in‐plane strains within the QD central plate, interestingly, the out‐of‐plane strain profiles show a locally different distribution in the growth direction. The piezoelectric polarization calculated using the measured in‐plane as well as out‐of‐plane strain indicates a significant reduction in the piezoelectric field compared to that of an equivalent planar heterostructure. The presence of the opposite sign of strain in the GaN QB and shell across the narrow inclined InGaN facet induces a dramatic reduction of the polarization charges and, therefore, a reduced piezoelectric field. This moderated piezoelectric field at the side facet of the InGaN QD can change the direction of the local piezoelectric field and increase the internal quantum efficiency.

## 4 Experimental Section

### Growth of Nanowire Heterostructure

Self‐assembled (In,Ga)N/GaN NWs were grown on Si (111) substrates by molecular beam epitaxy (MBE) as described in more detail in Refs. [23] and [40]. The NWs contain a superlattice of seven (In,Ga)N insertions in the GaN NWs. We note that the lowest of the insertions formed inadvertently during the growth interruption which was introduced to lower the substrate temperature. Below the superlattice, the NWs are at least 400 nm long, and on top of the superlattice, 30‐nm‐thick GaN cap was grown. The In content in the InGaN QD, the thickness of InGaN QD and GaN QB were determined by using X‐ray diffraction data via Bragg's law. On average, the In content is 30% and the thickness is 4.7 nm. The InGaN QDs are separated by the GaN barriers with the thickness of 14.6 nm.^[^ ^23^, ^40^ ^]^ The overview TEM image in Figure S1, Supporting Information, shows an ensemble with a high density of NWs that exhibit lengths of 500–700 and diameters of 50–130 nm.

### TEM Sample Preparation

For the conventional TEM imaging and DIH experiments, two different TEM samples were prepared. First, cross‐sectional TEM samples were prepared by the conventional mechanical dimpling method. The dimpled samples were ion‐milled using a 3 keV Ar^+^ ion beam (PIPS II, Gatan, Inc.) and later using a low energy Ar^+^ ion beam. For more detailed analyses of individual NWs, NW ensembles were mechanically detached from the substrate and directly transferred onto a lacey carbon TEM grid.

### Dark‐Field Inline Electron Holography

The DIH experiments were performed using a JEM‐2200F (Jeol Lt. Tokyo, Japan), equipped with a 200 kV field emission gun and an in‐column omega energy filter. All images were recorded using the energy filter to remove inelastically scattered electrons outside an energy window of +/−10 eV. An objective aperture of 10 µm in diameter was placed on the microscope's optical axis for selecting specific diffracted beams. Dark‐field TEM images at defocus values ranging from −8 to +8 µm were acquired exposing a 2048 × 2048 pixel fiber‐optically coupled UltraScan 1000 FT (Gatan, Inc.) camera for 6 s. Such dark‐field TEM focal series of a selected NW were recorded with the $\left(11\bar{2}0\right)$ and (0004) diffraction spots being selected (one focal series for each reflection). The obtained inline electron holograms were used to reconstruct the phase shift of the diffracted beam using the full resolution wave reconstruction (FRWR) algorithm.^[^ ^41^ ^]^ For the efficient sampling of high and low frequency information in the phase of exit wave function, the FRWR algorithm was used with incorporating the phase prediction function which uses a TIE‐like approach for the initial reconstruction of the high frequency phase information. In the strain mapping by DIH, the phase shift of the diffracted beam contains not only the geometric phase information (strain) but also the mean inner potential difference (Δ*V*_MIP_) along the direction of propagation. The Δ*V*_MIP_ between the InGaN and the GaN layer was 0.16 V (*V*_MIP_, _GaN_ = 16.82 V, *V*_MIP, InN_ = 17.35 V, *V*_MIP, In0.3Ga0.7N_ = 16.98 V). In the strain map the derivative of such a step function‐like Δ*V*_MIP_ across the interface introduces peaks at the interface. The calculation shows that the amplitude of the Δ*V*_MIP_‐induced peak is ignorable compared to the strain of the InGaN. Furthermore, if the interfaces are not perfectly sharp, as in the present case of the InGaN/GaN interfaces, the Δ*V*_MIP_‐induced phase shift across the interface is likely to be blurred out, further reducing its effect on the strain value.

### STEM Imaging and STEM‐EDS

STEM HAADF images were acquired using a JEM‐ARM200F microscope (Jeol Lt. Tokyo, Japan) equipped with a spherical aberration corrector (ASCOR, CEOS) operating at 200 kV accelerating voltage. The probe convergence angle of ≈36 and 32 mrad was used for HAADF and annular dark‐field (ADF) imaging, respectively. STEM ADF imaging mode combined with an energy dispersive X‐ray spectrometer (JED‐EDS, JEOL) was used to obtain elemental maps. A high‐resolution EDS map of the sample was acquired within several minutes by utilizing a dual EDS detector (each of them featuring an effective X‐ray sensing area of a 100 mm^2^) with a large effective solid angle of ≈0.8 sr and a highly focused electron probe (diameter ≈1.1 Å). The elemental maps were obtained by averaging up to 500 frames, sampled with 256 × 256 pixels and an acquisition time of 10 µs per pixel (≈5.5 min in maximum as a total acquisition time). For each of the maps the background noise floor was removed by Wiener filtering.

### 4D‐STEM

4D‐STEM images were acquired on a JEM‐ARM300F microscope (Jeol Lt. Tokyo, Japan) equipped with a spherical aberration corrector (COSMO, JEOL) operating at 300 kV accelerating voltage. The probe convergence angle of ≈8.3 mrad was used to avoid overlap between diffraction beams. The pixel resolution in real space image corresponds to ≈1 nm. Diffraction images were recorded using the 4096 × 4096 pixels CMOS camera (Gatan OneView camera, USA)

### FEM Simulation

Using the COMSOL Multiphysics software FEM within the framework of linear elasticity theory has been carried out for strain mapping. A 3D simulation cell was constructed by referring to the size and shape of the constituent layers of the NW heterostructure observed by direct TEM and STEM ADF images. For comparison with the experimental results, a hexagonal prism shape of the NWs with a uniform diameter over the entire length was assumed. For the In_0.3_Ga_0.7_N QDs the lattice constant and mechanical properties corresponding to the chemical composition were used. The bottom mount of the NW was fixed while no mechanical constraint was applied to the top and all the surrounding side surfaces, thus the strain was able to relax freely.

## Conflict of Interest

The authors declare no conflict of interest.

## Supporting information

Supporting Information

## Data Availability

The data that support the findings of this study are available from the corresponding author upon reasonable request.

## References

[1] I. Vurgaftman , J. R. Meyer , L. R. Ram‐Mohan , J. Appl. Phys. 2001, 89, 5815.

[2] S. Pereira , M. R. Correia , T. Monteiro , E. Pereira , E. Alves , A. D. Sequeira , N. Franco , Appl. Phys. Lett. 2001, 78, 2137.

[3] W. Shan , W. Walukiewicz , E. E. Haller , B. D. Little , J. J. Song , M. D. McCluskey , N. M. Johnson , Z. C. Feng , M. Schurman , R. A. Stall , J. Appl. Phys. 1998, 84, 4452.

[4] I. Ho , G. Stringfellow , Appl. Phys. Lett. 1996, 69, 2701.

[5] F. Glas , Phys. Rev. B 2006, 74, 121302.

[6] E. Ertekin , P. A. Greaney , D. C. Chrzan , J. Appl. Phys. 2005, 97, 114325.

[7] A. Akter , G. Yoo , S. Kim , H. W. Baac , J. Heo , J. Nanosci. Nanotechnol. 2017, 17, 3279.

[8] H. M. Kim , Y. H. Cho , H. Lee , S. I. I. Kim , S. R. Ryu , D. Y. Kim , T. W. Kang , K. S. Chung , Nano Lett. 2004, 4, 1059.

[9] T. Kehagias , G. P. Dimitrakopulos , P. Becker , J. Kioseoglou , F. Furtmayr , T. Koukoula , I. Häusler , a. Chernikov , S. Chatterjee , T. Karakostas , H.‐M. Solowan , U. T. Schwarz , M. Eickhoff , P. Komninou , Nanotechnology 2013, 24, 435702. 24076624 10.1088/0957-4484/24/43/435702

[10] X. Niu , G. B. Stringfellow , F. Liu , Appl. Phys. Lett. 2011, 99, 213102.

[11] G. Tourbot , C. Bougerol , F. Glas , L. F. Zagonel , Z. Mahfoud , S. Meuret , P. Gilet , M. Kociak , B. Gayral , B. Daudin , Nanotechnology 2012, 23, 135703. 22418250 10.1088/0957-4484/23/13/135703

[12] O. Marquardt , L. Geelhaar , O. Brandt , J. Comput. Electron. 2015, 14, 464.

[13] W. Sheng , J. P. Leburton , Phys. Rev. Lett. 2002, 88, 167401. 11955264 10.1103/PhysRevLett.88.167401

[14] J. Bartolomé , M. Hanke , D. Van Treeck , A. Trampert , Nano Lett. 2017, 17, 4654. 28735548 10.1021/acs.nanolett.7b01136

[15] M. Knelangen , M. Hanke , E. Luna , L. Schrottke , O. Brandt , A. Trampert , Nanotechnology 2011, 22, 469501. 10.1088/0957-4484/22/36/365703 21836335

[16] S. Y. Woo , N. Gauquelin , H. P. T. Nguyen , Z. Mi , G. A. Botton , Nanotechnology 2015, 26, 344002. 26234582 10.1088/0957-4484/26/34/344002

[17] J. Chung , L. Rabenberg , Ultramicroscopy 2008, 108, 1595. 18635317 10.1016/j.ultramic.2008.05.010

[18] Y. Zhu , C. Ophus , J. Ciston , H. Wang , Acta Mater. 2013, 61, 5646.

[19] M. Vallet , Y. Claveau , B. Warot‐Fonrose , C. Gatel , J. Nicolaï , N. Combe , C. Magen , R. Teissier , A. N. Baranov , A. Ponchet , Appl. Phys. Lett. 2016, 108, 211908.

[20] V. B. Özdöl , C. T. Koch , P. A. Van Aken , J. Appl. Phys. 2010, 108, 056103.

[21] K. Song , C. T. Koch , J. K. Lee , D. Y. Kim , J. K. Kim , A. Parvizi , W. Y. Jung , C. G. Park , H. J. Jeong , H. S. Kim , Y. Cao , T. Yang , L.‐Q. Chen , S. H. Oh , Adv. Mater. Interfaces 2015, 2, 1400281.

[22] J. K. Lee , B. Park , K. Song , W. Y. Jung , D. Tyutyunnikov , T. Yang , C. T. Koch , C. G. Park , P. A. van Aken , Y. M. Kim , J. K. Kim , J. Bang , L. Q. Chen , S. H. Oh , Acta Mater. 2018, 145, 109.

[23] M. Wölz , M. Ramsteiner , V. M. Kaganer , O. Brandt , L. Geelhaar , H. Riechert , Nano Lett. 2013, 13, 4053. 24001176 10.1021/nl401306q

[24] Z. Zhong , Q. P. Sun , Int. J. Solids Struct. 2002, 39, 5753.

[25] V. M. Kaganer , A. Y. Belov , Phys. Rev. B 2012, 85, 125402.

[26] K. Nishi , A. A. Yamaguchi , J. Ahopelto , A. Usui , H. Sakaki , J. Appl. Phys. 1998, 76, 7437.

[27] C. Pryor , J. Kim , L. W. Wang , A. J. Williamson , A. Zunger , J. Appl. Phys. 1998, 83, 2548.

[28] M. Tadić , F. M. Peeters , K. L. Janssens , M. Korkusiński , P. Hawrylak , J. Appl. Phys. 2002, 92, 5819.

[29] M. Korkusiński , P. Hawrylak , Phys. Rev. B 2001, 63, 195311.

[30] H. Shin , W. Lee , Y. H. Yoo , J. Phys. Condens. Matter 2003, 15, 3689.

[31] T. Krause , M. Hanke , O. Brandt , A. Trampert , Appl. Phys. Lett. 2016, 108, 032103.

[32] N. J. Ekins‐Daukes , K. Kawaguchi , J. Zhang , Cryst. Growth Des. 2002, 2, 287.

[33] B. Haas , J. L. Rouvière , V. Boureau , R. Berthier , D. Cooper , Ultramicroscopy 2019, 198, 58. 30660032 10.1016/j.ultramic.2018.12.003

[34] T. Grieb , F. F. Krause , K. Müller‐Caspary , R. Ritz , M. Simson , J. Schörmann , C. Mahr , J. Müßener , M. Schowalter , H. Soltau , M. Eickhoff , A. Rosenauer , Ultramicroscopy 2021, 228, 113321. 34175788 10.1016/j.ultramic.2021.113321

[35] E. P. O'Reilly , Semicond. Sci. Technol. 1989, 4, 121.

[36] D. Carvalho , K. Müller‐Caspary , M. Schowalter , T. Grieb , T. Mehrtens , A. Rosenauer , T. Ben , R. García , A. Redondo‐Cubero , K. Lorenz , B. Daudin , F. M. Morales , Sci. Rep. 2016, 6, 28459. 27350322 10.1038/srep28459 PMC4923855

[37] V. M. Kaganer , O. Marquardt , O. Brandt , Nanotechnology 2016, 27, 165201. 26963341 10.1088/0957-4484/27/16/165201

[38] O. Marquardt , L. Geelhaar , O. Brandt , J. Comput. Electron. 2015, 14, 464.

[39] O. Ambacher , B. Foutz , J. Smart , J. R. Shealy , N. G. Weimann , K. Chu , M. Murphy , A. J. Sierakowski , W. J. Schaff , L. F. Eastman , R. Dimitrov , A. Mitchell , M. Stutzmann , J. Appl. Phys. 1999, 87, 334.

[40] M. Wölz , V. M. Kaganer , O. Brandt , L. Geelhaar , H. Riechert , Appl. Phys. Lett. 2011, 98, 261907.

[41] C. T. Koch , Ultramicroscopy 2008, 108, 141. 17485172 10.1016/j.ultramic.2007.03.007

