# Supplementary material for: High‐Resolution Mapping of Strain Partitioning and Relaxation in InGaN/GaN Nanowire Heterostructures
Source: Adv Sci (Weinh). 2022 Jun 5;9(22):2200323. doi: 10.1002/advs.202200323 (PMC9353496; doi:10.1002/advs.202200323)
Supplement: Supplementary file 1 — Supporting Information [file ADVS-9-2200323-s001.pdf]

ADVANCED SCIENCE
Open Access

Supporting Information

for *Adv. Sci.*, DOI 10.1002/advs.202200323

High-Resolution Mapping of Strain Partitioning and Relaxation in InGaN/GaN Nanowire Heterostructures

*Bumsu Park, Ja Kyung Lee, Christoph T. Koch, Martin Wölz, Lutz Geelhaar and Sang Ho Oh**

Supporting Information

High-Resolution Mapping of Strain Partitioning and Relaxation in InGaN/GaN Nanowire Heterostructures

Bumsu Park[1, 2]*, Ja Kyung Lee*[1]*, Christoph T. Koch*[3]*, Martin Wölz*[4]*, Lutz Geelhaar*[4] *and Sang Ho Oh*[1,5*]

[1]Department of Energy Science, Sungkyunkwan University, Suwon 16419, Republic of Korea

[2]CEMES-CNRS, 29 rue J. Marvig, 31055 Toulouse, France

[3]Department of Physics, Humboldt University of Berlin, Berlin 12489, Germany

[4]Paul-Drude-Institut für Festkörperelektronik, Leibniz-Institut im Forschungsverbund Berlin e.V., Hausvogteiplatz 5-7, 10117 Berlin, Germany

[5]Department of Energy Engineering, KENTECH Institute for Energy Materials and Devices, Korea Institute of Energy Technology (KENTECH), Naju 58330, Republic of Korea

*e-mail: shoh@kentech.ac.kr

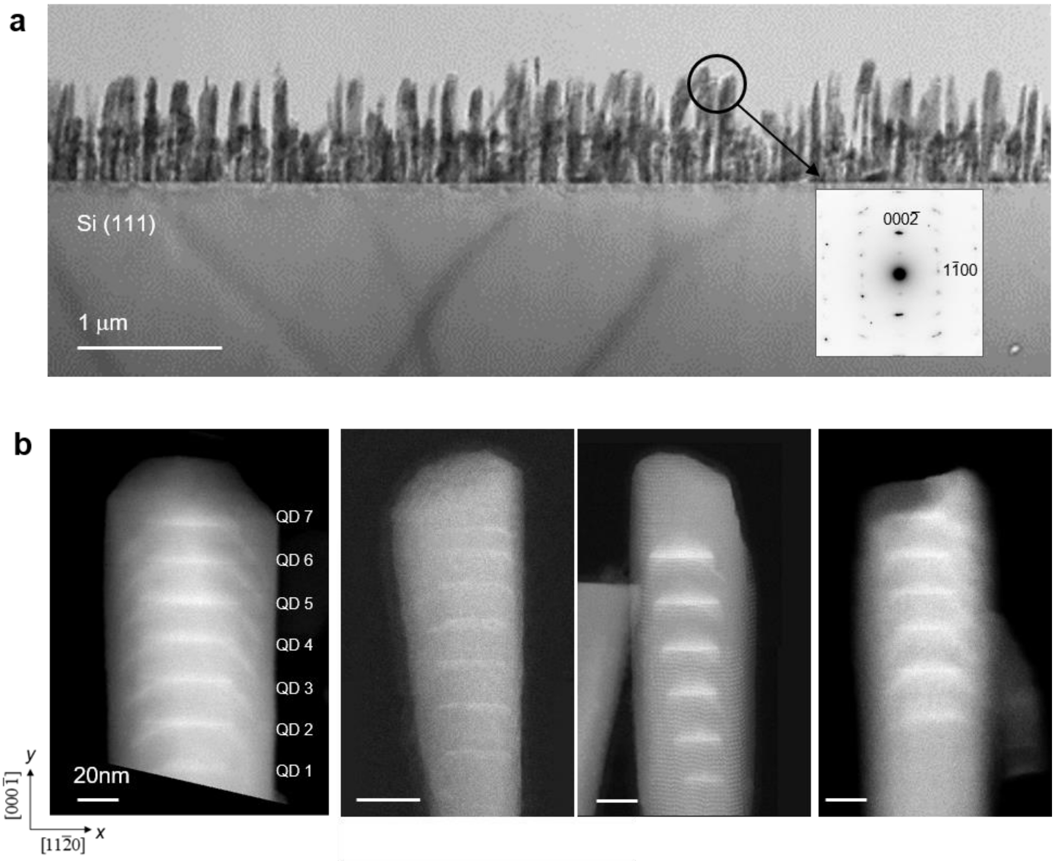

Figure S1. Axial InGaN/GaN NW heterostructures grown on Si (111) substrate. (a) TEM image and diffraction pattern of InGaN/GaN NW heterostructures. (b) STEM HAADF images of selected NWs. InGaN/GaN NWs were grown on Si (111) substrate. The InGaN QDs (brightest contrast in STEM-HAADF images) are fully embedded within the GaN NW. With increasing number of the QD stack, the side facets of the QDs become larger and steeper.

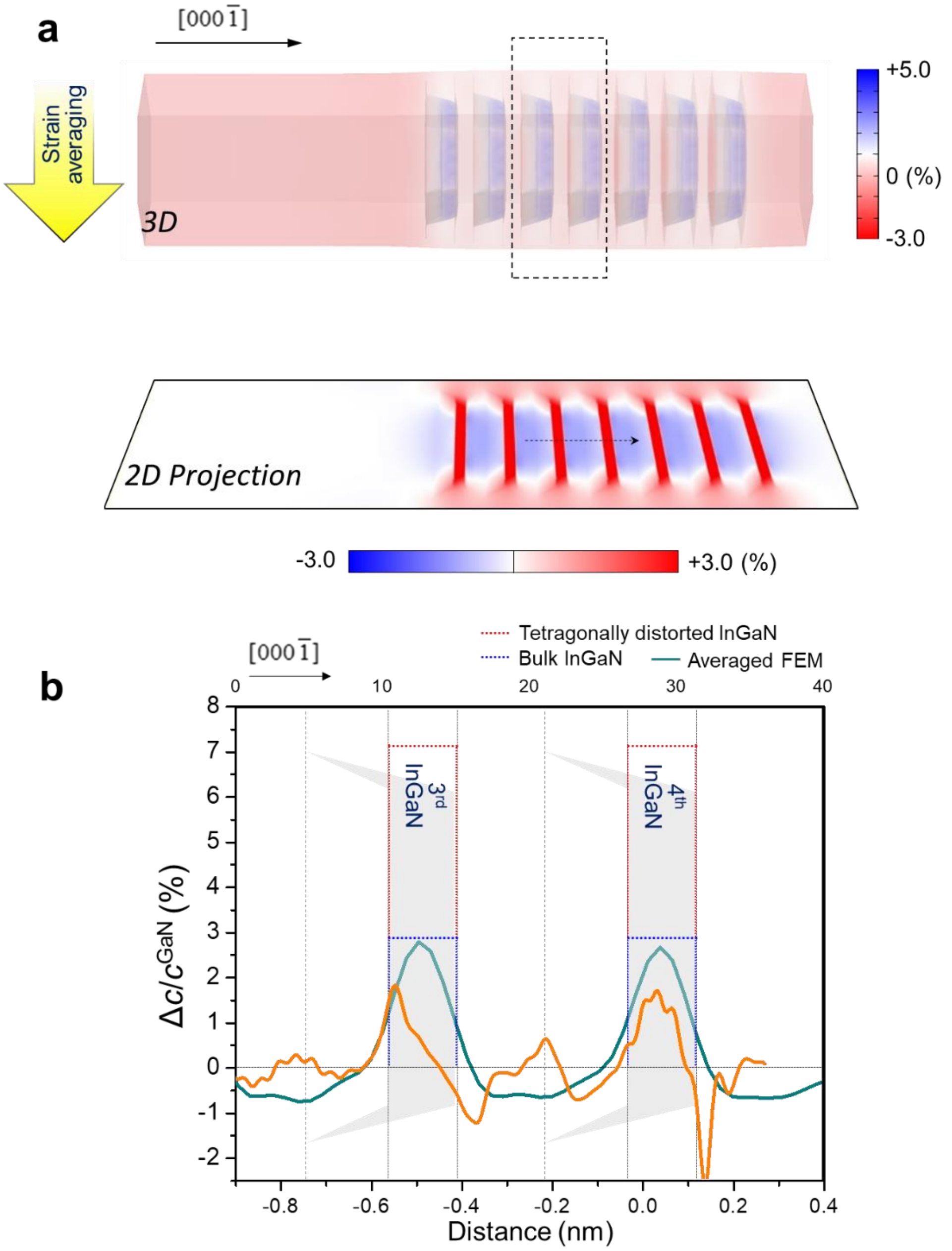

Figure S2. Projection of 3D FEM simulation result along the electron beam direction. (a) Schematic illustrating the concept of strain averaging of FEM 3D strain data along the electron beam direction to prepare 2D projected strain map for comparison with the experimental strain map. (b) Comparison of the out-of-plane strain profile obtained from DIH strain map (orange line) with the one from the 2D projected FEM strain map (cyan line). The strain expected for the bulk out-of-plane lattice parameter of InGaN is delineated by the blue-dotted line. The strain expected for the tetragonal distortion of InGaN due to the Poisson's effect is shown as red-dotted line. The two strain profiles agree well with each other.

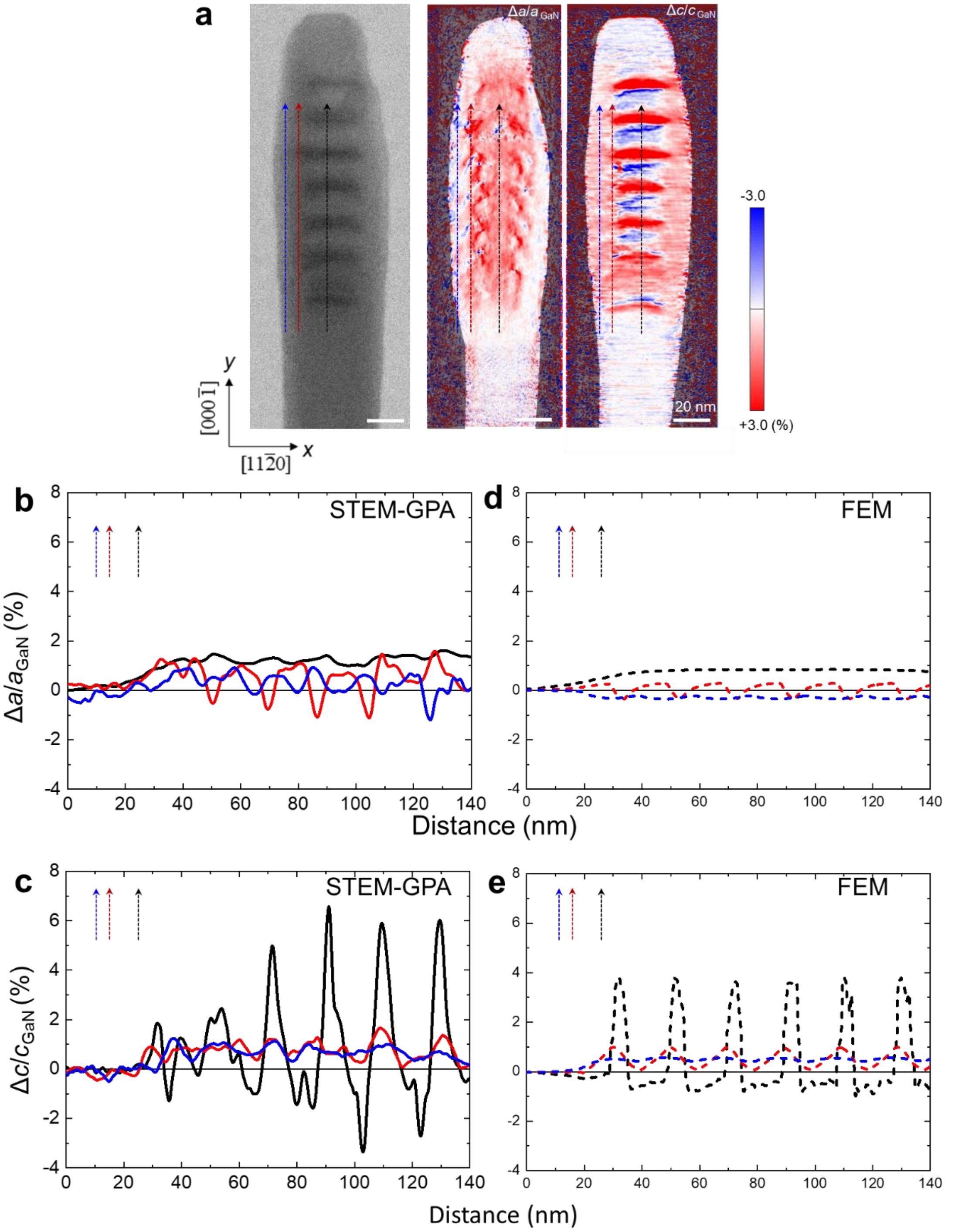

Figure S3. Strain profiles obtained from STEM GPA strain maps along the axial direction of the NW. (a) STEM BF image and corresponding in-plane and out-of-plane strain map obtained by STEM-GPA. Each arrow indicates the region from which the averaged strain profile was acquired (black: center of NW along the axial direction, red: the InGaN QD facet along the axial direction, blue: the GaN shell along the axial direction). The average width of each profile is 3 nm. (b) In-plane and (c) out-of-plane strain profiles from STEM-GPA and (d-e) FEM, respectively.

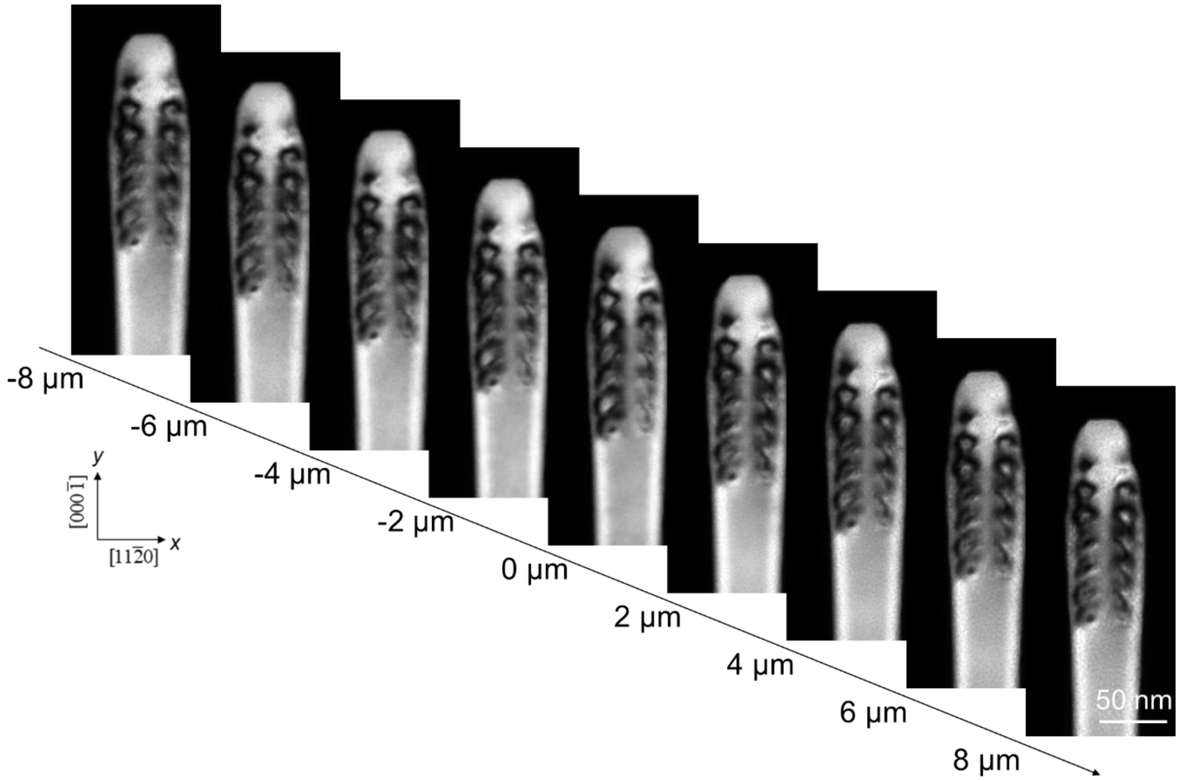

Figure S4. A focal series TEM DF images used for DIH. A typical through-focal data set for DIH were recorded with the $(11\bar{2}0)$ diffraction spots being selected. For one focal series nine TEM DF images in total were recorded at different defocus values ranging from -8 μm to +8 μm in 2 μm step.

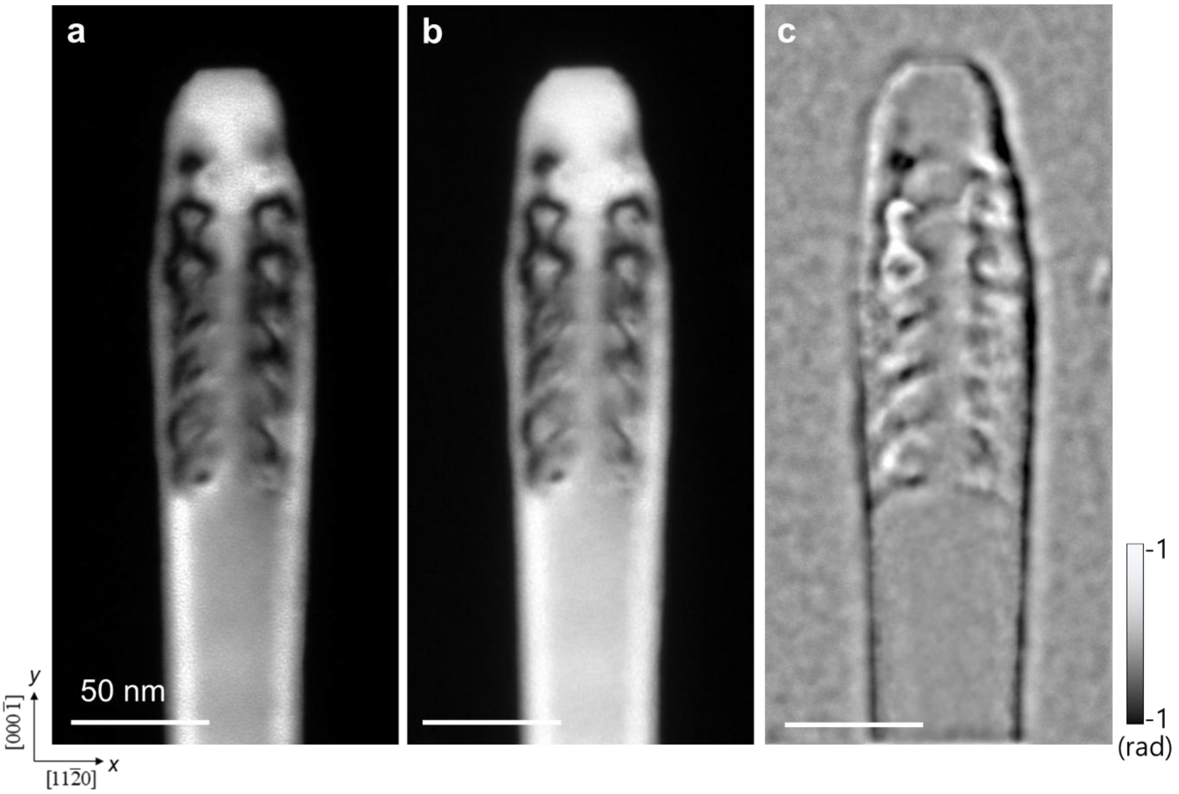

Figure S5. Phase and amplitude images reconstructed by using FRWR algorithm. (a) In-focus DF image, (b) amplitude image and (c) phase image of selected NW. 600 iterative reconstructions were done to obtain the final amplitude and phase images. Although very low spatial frequencies of the phase have not been fully recovered, computing the gradient of this phase for obtaining the strain map suppresses low spatial frequency information anyway.

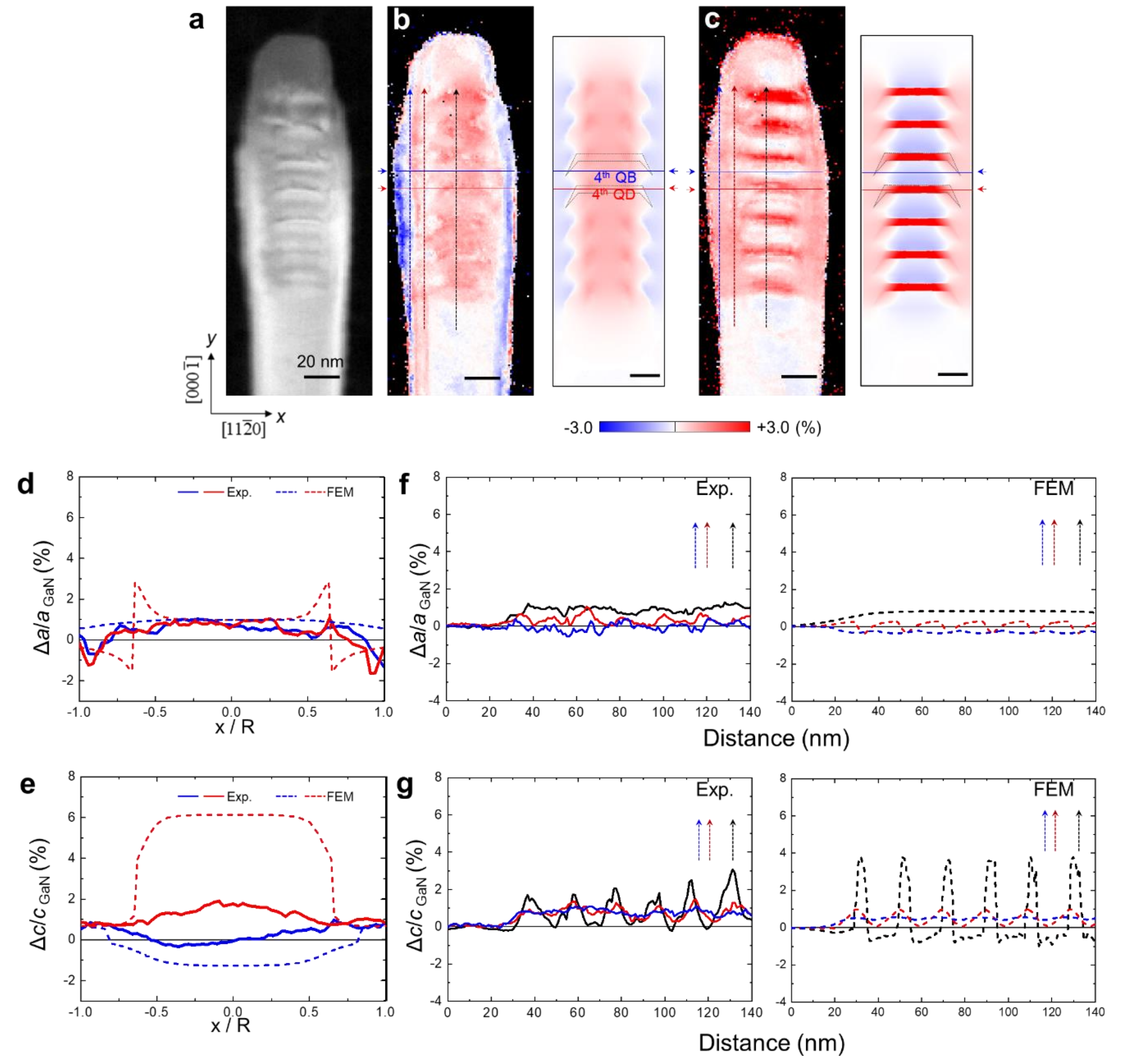

Figure S6. Strain mapping results of 4D-STEM. (a) STEM HAADF image of InGaN NW heterostructure chosen for 4D-STEM strain mapping. (b) In-plane and (c) out-of-plane strain maps obtained by 4D-STEM alongside with 3D FEM simulation results. Each arrow indicates the position from which the averaged strain profile was acquired (black dash line: center of NW along the axial direction, red dash line: center of InGaN facet along the axial direction, blue dash line: GaN shell along the axial direction, blue solid line: center of the 4th GaN QB along the radial direction, red solid line: center of the 4th InGaN QW along the radial direction). The average width of each profile is 3 nm. (d) Acquired in-plane and (e) out-of-plane strain profiles from the 4th InGaN QD and the 4th GaN QB region along the radial direction. (f) In-plane and (g) out-of-plane strain profiles along the axial direction from 4D-STEM (left) and FEM (right). Because of the limited spatial resolution of 4D-STEM technique, the measured strain values represent locally averaged strain values.

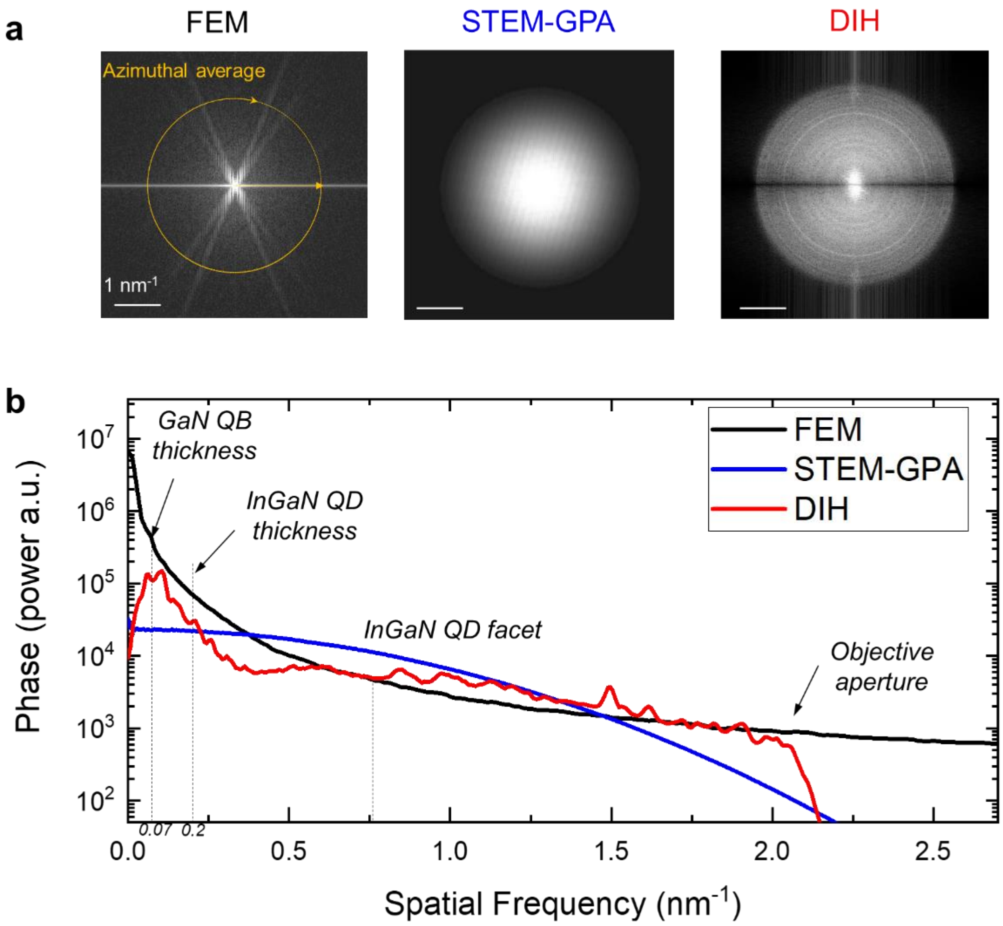

Figure S7. FFT of phase maps obtained by different techniques. (a) FFT of each phase map (FEM, STEM-GPA and DIH) and (b) azimuthally averaged profile of the power spectrum of the FFT of the phase map. The spatial resolution of STEM-GPA and DIH is comparable, which is below 0.5 nm. The length scales of various objects (the thickness of InGaN QD and GaN QB, the width of InGaN QD) are indicated. Compared with the STEM GPA, DIH shows better transmission of the high frequency information but poor transmission of the low frequency information.

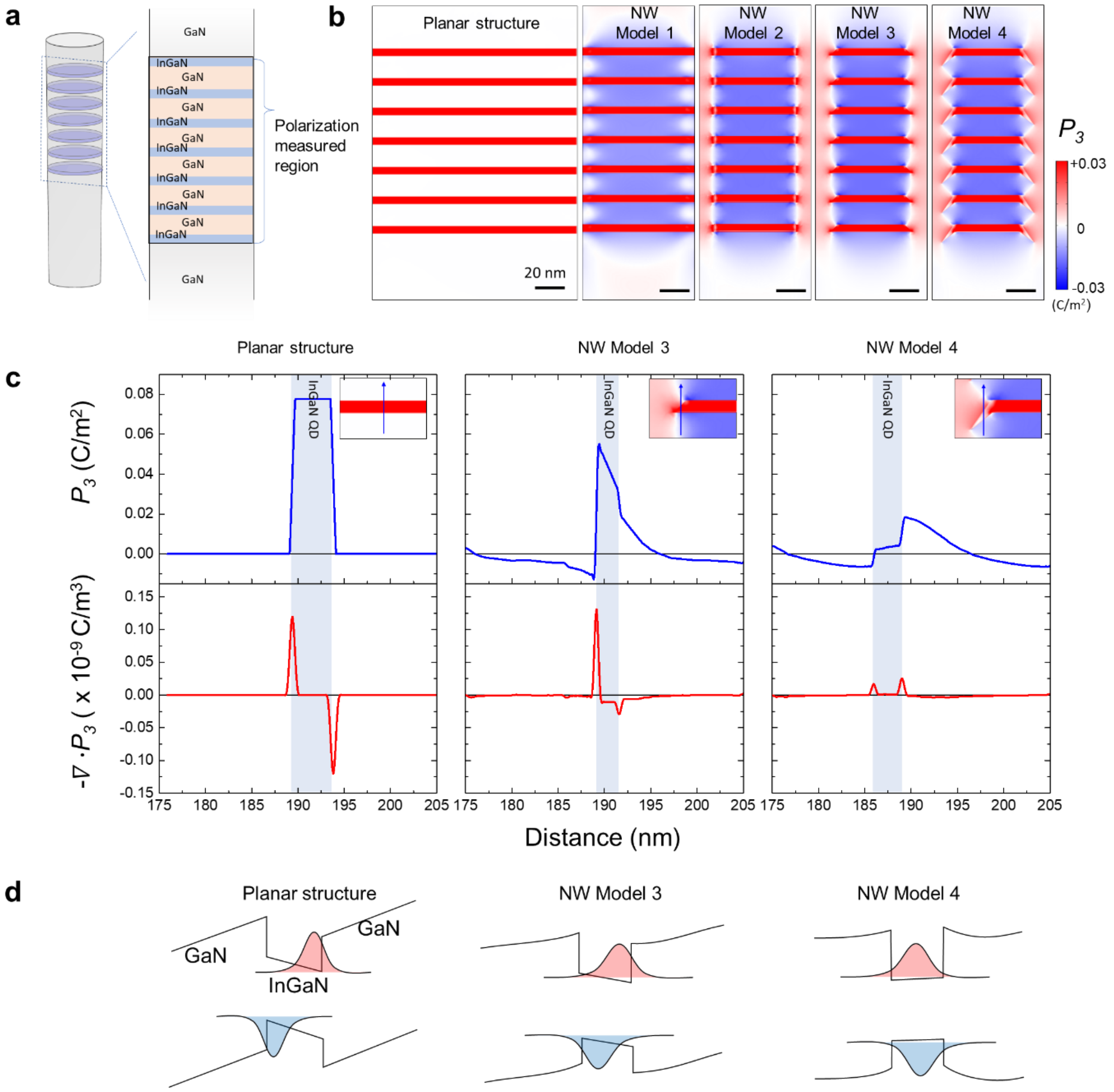

Figure S8. Piezoelectric polarization (P_3 component) calculated by using FEM strain results. (a) Schematic drawing of InGaN/GaN MQW region where the piezoelectric polarization has been calculated by using the strain data simulated by FEM. (b) 2D polarization maps of the four model NW heterostructures. The 2D P_3 component maps were extracted from the central region of from their respective 3D polarization maps. (c) 1D profile of P_3 polarization (upper panel) and gradient of P_3 (lower panel) obtained from the center of planar structure (left), the InGaN facet of NW model 3 (middle) and NW model 4 (right) along the axial direction. The facet of InGaN QD shows a lower polarization field than the planar structure. In particular, in the case of the QD with a truncated dome shape (NW model 4), the sign of polarization charge at the upper and the lower InGaN/GaN interfaces of the InGaN facet was the same, which results in a relatively flat band, i.e. smaller piezoelectric field, in the InGaN QD facet region. (d) Schematic illustration showing the band diagram and electron and hole wave function in InGaN QW of planar structure, NW model 3 and NW model 4.
